# Evolution of Jiang-Flavor Daqu’s Characteristics During Different Storage Stages and Influence on Simulated Brewing Fermentation

**DOI:** 10.3390/foods15020220

**Published:** 2026-01-08

**Authors:** Zihan Chen, Han Wang, Chongchao Wu, Xing Zheng, Guida Zhu, Jing Yu, Qiuxiang Tang, Ping Song

**Affiliations:** State Key Laboratory of Microbial Technology, School of Food Science and Pharmaceutical Engineering, Nanjing Normal University, Nanjing 210023, Chinajingyu@njnu.edu.cn (J.Y.)

**Keywords:** Jiang-flavor Daqu, storage period, microbial community, volatile flavor compounds, simulated brewing fermentation, untargeted metabolomics

## Abstract

Daqu quality plays a crucial role in the entire fermentation process of Baijiu. There is no empirical evidence for a scientific consensus on the storage period of Jiang-flavor Daqu and its quality evaluation. This study took Jiang-flavor Daqu from a liquor enterprise in Sichuan Province as the research object. It explored the changes in physicochemical indexes, microbial communities, and volatile flavor substances of the Daqu within 0–180 days of storage. Combined with simulated brewing experiments, it analyzed the effects of different storage periods of Daqu on fermented grain fermentation and the base wine quality and clarified the metabolic differences between Daqu stored for 30 days and 180 days by means of metabolomics. The results showed that the saccharification power and fermentation power of Daqu first increased and then stabilized, reaching 205 mg/g·h and 0.71 g/g·72, respectively, at 180 days. The microbial diversity first increased and then decreased, with *Virgibacillus* and *Oceanobacillus* alternately serving as the dominant bacteria. The flavor substances were more abundant within 60 days of storage, while the content of pyrazine compounds was the highest at 180 days. The wine yield of Daqu stored for 30 days was 2.26 times that of Daqu stored for 180 days. The brewing stage had the greatest impact on metabolites, and flavonoid synthesis was the key metabolic pathway. This study provides theoretical support for the scientific storage of Jiang-flavor Daqu and the standardization of its quality.

## 1. Introduction

Baijiu, a globally recognized representative distilled liquor, is also a core carrier of China’s traditional brewing culture. Its stable and substantial annual output not only reflects the industrial scale but also solidifies its important position in the global spirits market [1]. The industry employs over 2 million people directly and supports numerous agricultural communities through grain procurement, with the annual consumption of sorghum, wheat, and corn exceeding 20 million tons. This substantial economic impact establishes Baijiu’s dual importance as both intangible cultural heritage and an economic pillar in China [2]. Daqu production uses wheat as the main raw material, supplemented by barley and peas [3]. Mixed with water and high-quality Daqu powder from the previous year, it is pressed into bricks, spontaneously fermented at 50–60 °C for 25–30 days, then stored for 3–6 months until moisture reaches 15% and temperature stabilizes. During fermentation, starch hydrolases and other enzymes aid flavor formation; dominant thermotolerant microbes like *Bacillus* and *Thermomyces* function by producing specific enzymes. Open fermentation further fosters a complex, stable microbial community [4]. Moreover, the stable maintenance of this industrial scale hinges on Daqu—an indispensable core fermentation starter in the Baijiu brewing system. The traditional saying, “Good Qu produces good wine,” is a classic summary of the inherent connection between the two [5]. Daqu’s own quality not only directly determines the overall standard of finished Baijiu but also serves as the fundamental support for shaping the core quality characteristics of Baijiu [6]. As a key material supply source for the formation of Baijiu quality, the quality of Daqu directly affects the final quality of Baijiu.

The Daqu storage period directly affects Daqu quality and serves as a key stage for shaping Daqu quality, which in turn determines the liquor quality. However, current Daqu storage durations vary significantly, and no scientific conclusion has been reached yet. Additionally, there is insufficient analysis of the internal correlation mechanism between the storage period and Daqu quality, and unified storage standards are lacking. Therefore, exploring the mechanism by which the storage period influences Daqu quality is imperative.

Several studies have explored the storage period’s impact on Daqu quality. Zhou studied strong-flavor Daqu, finding that key enzyme activities (saccharifying, liquefying, etc.) peaked at 3–4 months of storage; dominant bacterium *Weissella* increased in abundance while dominant fungus *Thermomyces* decreased, and this period suited Baijiu brewing best [7]. Yang noted that for Jiang-flavor Daqu, *Bacillus*, *Oceanobacillus*, etc., increased significantly during storage, while *Kroppenstedtia*, etc., decreased, and metabolism shifted from small-molecule production in the first 3 months of storage to amino sugar metabolism later [8]. Ren found that for strong-flavor Jinhui Daqu, Daqu stored for 8 days had higher fungal diversity than 45-day and 90-day ones, while Daqu stored for 90 days had the highest bacterial diversity, with microbial structure differences causing 993 differential metabolites [9]. Guan defined optimal storage periods for two strong-flavor Daqu types (M-Daqu: 60–210 days; T-Daqu: 60–180 days) [10]. Yet, all these studies only focus on the storage period’s impact on Daqu, lacking mechanistic research on how microorganisms influence Daqu quality and further fermentation.

For consumers, the sensory experience of Jiang-flavor Baijiu—featuring roasted, nutty, mellow aromas and a harmonious taste—is the core criterion for a product quality evaluation, and these traits are directly shaped by Daqu quality [11]. However, the absence of unified Daqu storage standards results in an inconsistent sensory quality of finished Baijiu, confusing consumers. Regulatorily, China has formulated national standards (e.g., GB/T 10781.4-2024) [12] for Jiang-flavor Baijiu, specifying finished product indicators, but lacks unified national/industry standards for Daqu storage periods, hampering quality traceability and regulatory supervision [13]. As a core carrier of Chinese traditional brewing culture, the inheritance and development of Jiang-flavor Baijiu depend on standardizing key links like Daqu storage—critical for preserving the authenticity of traditional flavors and cultural connotations [2].

To address the above issues, this study focuses on Jiang-flavor Daqu from a Sichuan liquor enterprise, aiming to systematically explore the dynamic changes in physicochemical indexes, microbial communities and volatile flavor substances of Daqu within 0–180 days of storage; clarify the effects of different storage periods on fermented grain fermentation and base wine quality through simulated brewing experiments; reveal the metabolic differences between Daqu stored for 30 days and 180 days via untargeted metabolomics; and establish a scientific basis for the standardized storage of Jiang-flavor Daqu. This research is expected to meet the consumer demand for stable sensory quality, comply with relevant national regulatory requirements, and provide theoretical support for the inheritance and standardized development of traditional Jiang-flavor Baijiu culture.

## 2. Materials and Methods

### 2.1. Sample Collection and Simulated Fermentation

The Jiang-flavor Daqu used in this study was sourced from a Baijiu enterprise in Sichuan Province. Three pieces of Daqu were sampled from the upper, middle, and lower layers of each Daqu-making room, with samples collected from 3 rooms and then mixed uniformly [6]. The samples were labeled sequentially as 0 d, 15 d, 30 d, 60 d, 90 d, 120 d, 150 d, and 180 d, in chronological order. Both crushed sorghum and bran husks were provided by the same Baijiu enterprise in Sichuan Province. Samples collected during the brewing stage included those taken pre-stacking, at pit-entry, and at pit-exit, which were labeled as 0 d, 30 d, 60 d, 90 d, 120 d, 150 d, and 180 d, respectively [14].

A total of 8 kg sorghum was crushed into 4–6 pieces (pre–soaked for 12 h with water at 50% of its mass) and mixed with bran husks at a ratio of 10:1. We added 10% (of sorghum mass) Jiang-flavor Daqu of different storage periods (crushed through 40-mesh sieve), mixed well, then heap-fermented it at 30–35 °C, 60–65% humidity for 2–3 days (1–2 turnings) [14]. We transferred it to sealed fermenters and fermented it for 30 days with 3 replicates per group, sampling regularly. Daqu is also named “quyao” (QY). The samples collected during the brewing stage were the fermented grains (zaopei) before heaping (DJ), upon pit entry (RJ), and upon pit exit (CJ), respectively.

### 2.2. Analysis of Physicochemical Indexes

The determination of the basic physicochemical indices of Daqu (moisture, acidity, and starch), the quality indices of Daqu (saccharifying power, fermenting power, and esterifying power), and the physicochemical indices (moisture, acidity, starch, and reducing sugar) in fermented grain samples collected at the pre-stacking, pit-entry, and pit-exit stages were all conducted in accordance with QB/T 4257-2011 General Analytical Methods for Distiller’s Daqu [15].

### 2.3. High-Throughput Sequencing of Daqu

The full-length amplification primers for bacterial 16S rDNA were 338F and 806R, while the amplification primers for the fungal ITS region were ITS1 and ITS2 [16]. The PCR amplification reaction system contained 1 μL of template DNA, 1 μL each of the forward and reverse primers, 25 μL of 2× Taq PCR Master Mix, and 22 μL of double-distilled water [14,17]. The reaction conditions were set as follows: pre-denaturation at 94 °C for 3 min, followed by 30 cycles of denaturation at 94 °C for 30 s, annealing at 55 °C for 45 s, and extension at 72 °C for 45 s, with a final extension at 72 °C for 10 min [18]. The amplified sequences were sequenced and analyzed by Shanghai Majorbio Bio-pharm Technology Co., Ltd. (Shanghai, China).

### 2.4. Determination of Volatile Flavor Compounds

Volatiles in Jiang-flavor Daqu were analyzed via HS-SPME-GC-MS. Briefly, 1 g of homogenized Daqu powder was placed in a headspace vial, spiked with 5 μL 2-octanol (internal standard), equilibrated at 60 °C for 5 min, headspace-adsorbed for 45 min, and desorbed at 250 °C for 5 min before GC-MS analysis [15]. GC was conducted with a DB-WAX capillary column (30 m × 250 μm × 0.25 μm, Agilent, Santa Clara, CA, USA), an injector temperature of 250 °C (splitless injection), and high-purity helium as carrier gas (1.0 mL/min). MS was operated in EI mode (70 eV), ion source temperature 230 °C, and full-scan range 40–400 amu. Unknown compounds were identified by library matching (match similarity > 85%), semi-quantified using 2-octanol as the internal standard, and flavor substance concentrations were calculated per Reference [19].

### 2.5. Metabolomics Analysis of Daqu Microorganisms

An adequate quantity of the sample was taken, weighed precisely into a 2 mL centrifuge tube, and mixed with 600 µL of methanol. This methanol, stored at −20 °C, contained 4 ppm 2-amino-3-(2-chlorophenyl) propionic acid. Then, 100 mg of glass beads were added, and the tube was placed in a tissue grinder for 90 s of grinding at 60 Hz [20]. After grinding, the mixture was centrifuged at 12,000 rpm and 4 °C for 10 min. The supernatant was filtered through a 0.22 μm membrane filter and transferred to a detection vial for LC-MS analysis [21]. Partial least squares discriminant analysis (PLS-DA) analysis of Daqu metabolic data was carried out, using SIMCA software 14.1 (Umetrics, Sweden) [22]. Metabolic pathway analysis was conducted via Metabo Analyst Version 5.0, accessible at https://www.metaboanalyst.ca/. Volcano plots showing the classification of differential metabolites were constructed with the gg volcano R package (Version 1.3.2, retrieved from https://CRAN.R-project.org/package=ggvolcano (accessed on 5 November 2025) [23].

### 2.6. Statistical Analysis

Sampling sketches were created with Adobe Illustrator 2021. Graphs for visualizing trends in Daqu’s physicochemical indicators were generated via GraphPad Prism 9.5. For assessing the alpha and beta diversity of samples—including Chao1 and Shannon indices—the vegan package within R software (Version 4.1.1, accessible at https://www.r-project.org/) was employed [24,25]. Redundancy analysis was carried out via the same vegan package. Prior to this analysis, the microbial dataset underwent transformation using the Hellinger method, whereas the physicochemical dataset was transformed using the Log method. Analysis of variance (ANOVA) and Duncan’s test were conducted using SPSS Version 21 to evaluate differences, with *p* < 0.05 being regarded as statistically significant [15]. Prior to ANOVA analysis, data normality was assessed using the Shapiro–Wilk test (n < 50) and homogeneity of variances was evaluated using Levene’s test. For datasets that violated normality assumptions (*p* < 0.05), appropriate transformations (log or square root) were applied. When transformations failed to achieve normality, non-parametric alternatives (Kruskal–Wallis test) were employed. Statistical significance was set at *p* < 0.05.

## 3. Results and Discussion

### 3.1. Physicochemical Differences in Jiang-Flavor Daqu at Different Storage Stages

**Figure 1:**
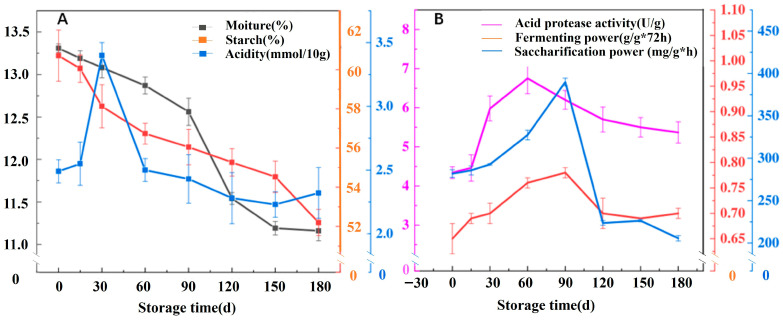
Moisture, acidity, starch (**A**), saccharification power, fermentation power, and acidic protease (**B**) of Jiang-flavor Daqu at different storage periods.

Physicochemical indexes, including basic indexes (moisture, starch, and acidity) and quality-related indexes (saccharification power, fermentation power, and acidic protease activity), were determined to evaluate the maturity and quality stability of Jiang-flavor Daqu during 0–180 days of storage. The moisture content decreased continuously with extended storage, while the starch content declined gradually, which was attributed to microbial decomposition for flavor substance synthesis. Acidity increased significantly in the early storage stage (0–90 days) and stabilized after 120 days, which was associated with dynamic microbial metabolic activity (Figure 1A). For quality indexes, saccharification power and fermentation power first increased and then stabilized (Figure 1B). At 180 days, saccharification power and fermentation power reached 205 mg/g·h and 0.71 g/g·72 h, respectively. Acidic protease activity increased with prolonged storage, especially after 90 d, which was possibly due to the temperature adaptation of protease-producing microorganisms. Proteases can hydrolyze proteins into amino acids, and the decomposition products of amino acids can provide aroma substances for Baijiu. The activity of acid proteases increases with the rise in temperature, which also explains why the content of acid proteases keeps increasing after 90 days of storage [26]. Saccharification power showed a distinct peak at 90 days (198 mg/g·h) before stabilizing at 180 days (205 mg/g·h). This pattern reflects the succession of amylase-producing microorganisms. Over 0–90 days, thermophilic bacteria (particularly *Bacillus*) and fungi (*Aspergillus*, *Thermomyces*) actively proliferate and secrete α–amylase and glucoamylase. The slight decrease at 120 days may result from enzyme degradation and reduced microbial metabolic activity as nutrients become depleted. The subsequent recovery and stabilization at 150–180 days indicate the establishment of a balanced microbial ecosystem where enzyme production and degradation reach equilibrium. This interpretation is supported by the microbial diversity data showing maximum bacterial diversity at 120 days.

### 3.2. Microbial Community Diversity of Jiang-Flavor Daqu with Different Storage Periods

High-throughput sequencing of the bacterial 16S rRNA gene (primers: 338F/806R) and fungal ITS region (primers: ITS1/ITS2) was performed on Jiang-flavor Daqu samples with different storage periods (0, 15, 30, 60, 90, 120, 150, and 180 days) from a Sichuan liquor enterprise, and data were analyzed using the QIIME and R packages [27]. After quality control, a total of 261,469 to 268,754 valid 16S rRNA sequences were obtained per bacterial sample, with an average length of 426 bases, and 155,179 to 205,509 valid ITS sequences per fungal sample, with an average length of 243 bases. Sequences sharing ≥97% similarity were clustered into operational taxonomic units (OTUs), resulting in 2464 bacterial OTUs and 335 fungal OTUs. The dilution curves of bacterial and fungal communities gradually flattened, and the coverage values of all samples exceeded 99%, indicating sufficient sequencing depth to capture the microbial diversity of Daqu [28,29]. Alpha diversity indices were calculated: the ACE (abundance-based coverage estimator) and Chao1 index for richness estimation, the Shannon diversity index for combined richness and evenness, and the Simpson diversity index for dominance patterns. Bacterial communities showed the maximum richness at 120 days (ACE = 431.77, Chao1 index = 430.88) and the highest diversity (Shannon index = 2.90, Simpson index = 0.91). Fungal richness reached the highest at 150 days (ACE = 128.50, Chao1 = 131.07) and diversity was highest at 180 days (Shannon = 2.25), suggesting dynamic adjustment and gradual balancing of microbial communities in the late storage stage (Table 1).

**Figure 2:**
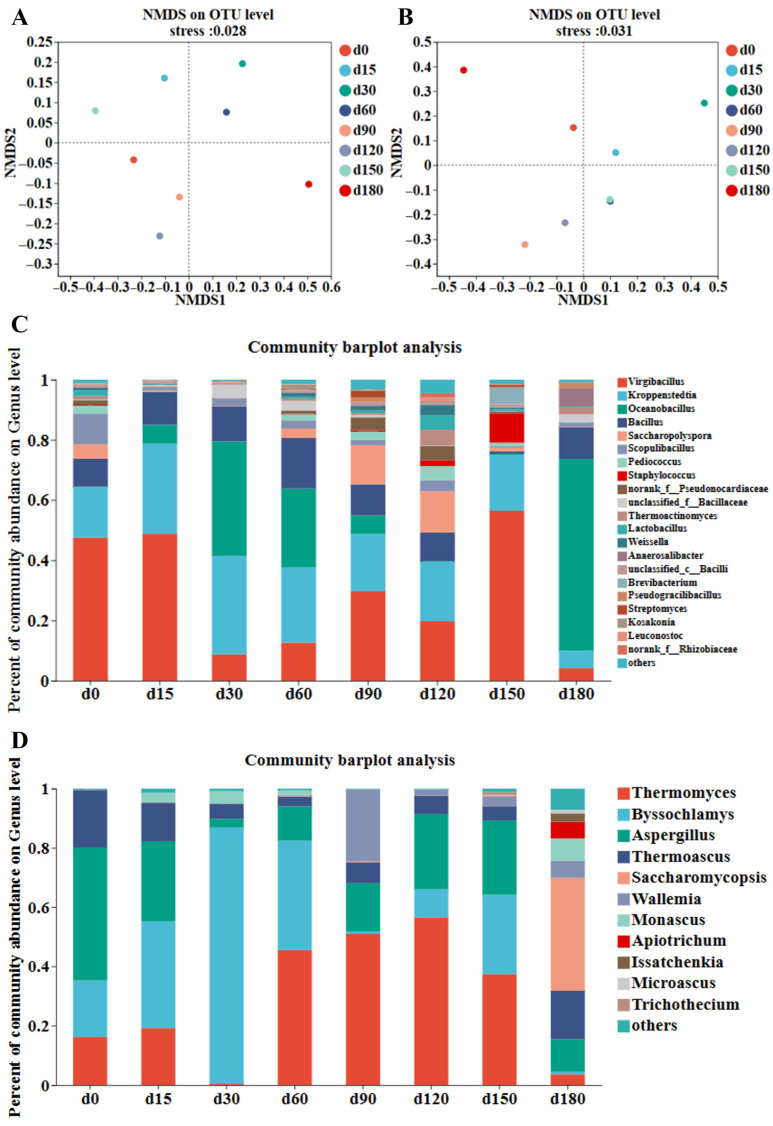
Analysis of microbial communities in Jiang-flavor Daqu with different storage periods. NMDS analysis of (**A**) bacterial communities and (**B**) fungal communities of Jiang-flavor Daqu at different storage stages. (**C**) Bacterial communities at the genus level of Jiang-flavor Daqu at different storage periods. (**D**) Fungal communities at the genus level of Jiang-flavor Daqu at different storage periods.

Non-metric multidimensional scaling (NMDS), based on Bray–Curtis distance, showed clear community separation (bacterial stress = 0.028, fungal stress = 0.031). Bacterial communities of Daqu stored for 30, 60, 90, and 120 days had high similarity, while fungal communities were widely distributed across quadrants, indicating significant differences between storage periods (Figure 2A,B). Hierarchical clustering analysis further confirmed this pattern, with bacterial samples from adjacent storage periods clustering together and fungal samples showing distinct separation (Figure S1).

At the phylum level, three dominant bacterial phyla (Firmicutes, Actinobacteriota, and Proteobacteria) and two fungal phyla (Ascomycota and Basidiomycota) were identified (Figure S2). Firmicutes was the absolutely dominant bacterial phylum, with its relative abundance gradually increasing and maintaining a high level in the middle and late storage stages; Actinobacteriota and Proteobacteria had relatively low abundances and showed no obvious fluctuation [30,31]. For fungi, Ascomycota was the dominant phylum, with relative abundance exceeding 90% across all samples, while Basidiomycota had extremely low abundance (less than 1%) and no significant change with storage time. At the genus level, dominant bacterial genera included *Virgibacillus*, *Kroppenstedtia*, *Bacillus*, and *Oceanobacillus*. *Virgibacillus* showed absolute dominance in Daqu stored for 0, 15, 60, 90, and 120 days, while *Oceanobacillus* became the dominant genus at 30, 60, and 180 days, presenting an alternating dominance pattern (Figure 2C). *Kroppenstedtia* was positively correlated with acidic protease activity. Studies have shown that *Kroppenstedtia* is a core genus involved in fatty acid biosynthesis during the brewing process, contributing to the production of fatty acids [32]. As fatty acids are important aroma and flavor components of Jiang-flavor Baijiu, this indicates that *Kroppenstedtia* is crucial for enhancing the flavor of Baijiu [8]. It can increase the content of substances such as acetic acid during the fermentation process. *Bacillus* can produce amylase and protease to promote the formation of flavor precursors such as 2,3,5,6-tetramethylpyrazine [33]. Dominant fungal genera shifted sequentially with the storage period. *Aspergillus* was dominant at 0 days. *Byssochlamys*, which is capable of degrading starch and cellulose in raw materials, replaced *Aspergillus* as the main genus at 15–30 days (Figure 2D). *Thermomyces*, which has heat resistance and the ability to produce hydrolytic enzymes that assist saccharification and fermentation, became dominant at 60–150 days. *Saccharomycopsis* took the leading position at 180 days. *Thermomyces* and *Thermoascus* are common in the fungal community of Jiang-flavor Daqu. They can produce a variety of thermostable hydrolases to assist stacking saccharification and fermentation, and thus promote the formation of Jiang-flavor characteristics. *Thermomyces* and *Thermoascus* can produce multiple types of enzymes, and they can maintain stable activity at relatively high fermentation temperatures [8]. The hydrolases produced by *Aspergillus* act on the processes of starch saccharification, protein hydrolysis, and flavonoid production. Previous studies on traditional fermented foods have confirmed that climatic and seasonal factors affect the structure and function of microbial communities in the substrate, and that microbial communities with longer storage periods are more significantly affected by these factors [23]. In summary, the microbial community diversity of Jiang-flavor Daqu changed dynamically with the storage period and tended to stabilize in the late stage, which is crucial for maintaining Daqu quality and subsequent brewing performance [6,34].

### 3.3. Volatile Flavor Substances of Jiang-Flavor Daqu with Different Storage Periods

**Figure 3:**
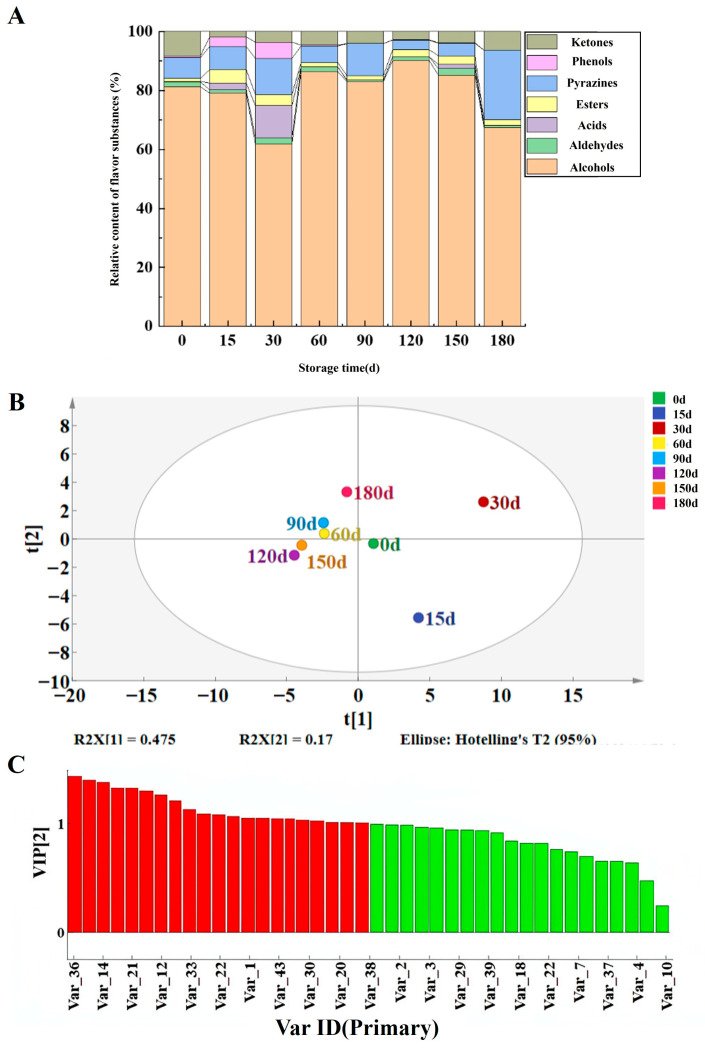
Study on volatile compounds in Daqu with different storage periods. (**A**) Relative content of volatile flavor substances of Jiang-flavor Daqu at different storage periods. (**B**) PLS–DA score plot of Jiang-flavor Daqu metabolites at different storage periods. (**C**) VIP diagram of important metabolites.

Headspace solid-phase microextraction coupled with gas chromatography–mass spectrometry (HS-SPME-GC-MS) was used to analyze volatile flavor substances in Jiang-flavor Daqu with different storage periods (0, 15, 30, 60, 90, 120, 150, and 180 days) from a Sichuan liquor enterprise. A total of 82 volatile compounds were identified, belonging to eight classes: 21 alcohols, 15 esters, 13 pyrazines, 11 ketones, 7 organic acids, 7 aldehydes, 5 phenols, and 3 others. With the increase in storage period, aldehydes and ketones remained relatively stable. Acids, esters, and phenols all showed a trend of first increasing and then stabilizing. Pyrazines gradually increased, reaching the maximum relative content of flavor when stored for 180 days (Figure 3A).

The volatile profile varied dynamically with storage time. Daqu stored within 60 days had richer flavor substances, such as isoamyl alcohol (peaked at 0.415 μg/g at 30 days), 4-ethylguaiacol (peaked at 0.232 μg/g at 15 days), and 2-methylpyrazine (Figure S3). In the late storage stage, off-odor compounds (e.g., octanoic acid, propanoic acid) decreased, while pyrazines—key aroma contributors to Jiang-flavor Baijiu—accumulated gradually, with 2,3,5,6-tetramethylpyrazine and 2,3,5-trimethylpyrazine reaching their highest contents (0.977 μg/g and 0.344 μg/g, respectively) at 180 days. PLS-DA clearly distinguished Daqu across storage periods (Figure 3B). Thirty-two compounds with variable importance in projection (VIP) > 1 (e.g., 4-ethylguaiacol, phenylethanol, 2,3,5-trimethylpyrazine) were identified as potential biomarkers (Figure 3C and Table S1). Pyrazines are important flavor-active components in Jiang-flavor Baijiu [26]. They can directly contribute typical Jiang-flavor style aromas, such as burnt aroma, nutty aroma, and roasted aroma, and also coordinate the flavor layers of the liquor body and enhance flavor recognition, playing a key supporting role in the formation of the unique flavor of Jiang-flavor Baijiu [35]. In the late phase of Daqu storage, amino sugar metabolism becomes the primary metabolic process of Daqu, which also acts as a survival strategy for microbes in oligotrophic environments [36]. This indicates that the directly utilizable carbohydrates in Daqu are insufficient to facilitate microbial proliferation during this late storage phase, and the Daqu thereby enters a stable state [8]. Esters form through enzymatic esterification between alcohols and organic acids catalyzed by esterases from yeasts and bacteria. The esterification reaction catalyzed by ester synthase using alcohols and acids as the substrates is usually recognized as the main pathway to synthesize fatty acid ethyl esters in strong-flavor Baijiu [37]. Through the esterification process between carboxylic acids and alcohols, esterases are capable of generating aromatic esters that enhance the synthesis of flavor compounds in Daqu, consequently influencing the quality of the resulting Baijiu [38]. During fermentation, esterases directly catalyze the esterification of acetic and propionic acids with ethanol to synthesize ethyl acetate and ethyl propionate [39]. Organic acids result from carbohydrate fermentation through glycolysis and the TCA cycle. The TCA cycle, an important metabolic pathway in Baijiu fermentation, generates organic acids such as oxalic acid, citric acid, isocitric acid, succinic acid, and malic acid, contributing to the characteristic sour and umami flavors, and it also produces L-glutamic acid, which can be further converted into D-(−)-glutamine, proline, and hypoxanthine, contributing to the development of bitterness [40].

### 3.4. Differential Analysis of Microbial Metabolomics in Different Seasons of Daqu

**Figure 4:**
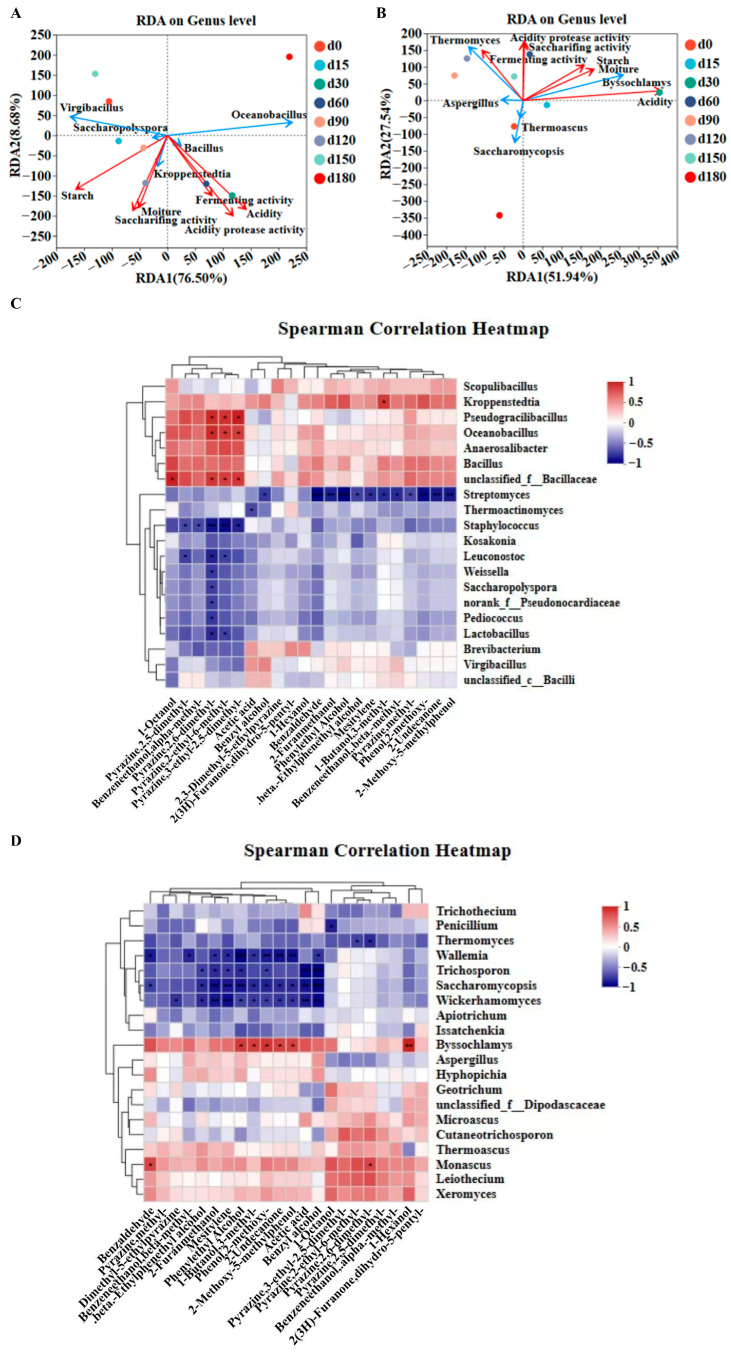
Study on relationships among microbial communities, physicochemical properties, and volatile flavor substances of Jiang-flavor Daqu at different storage stages. RDA of (**A**) bacterial community and (**B**) fungal community and physicochemical indexes. (**C**) Spearman analysis of bacterial communities and volatile flavor substances. (**D**) Spearman analysis of fungal communities and volatile flavor substances. * indicates *p* < 0.05, ** indicates *p* < 0.01, and *** indicates *p* < 0.001.

Redundancy analysis (RDA) and Spearman correlation analysis were conducted to explore the associations between microbial communities, physicochemical indexes, and volatile flavor substances of Jiang-flavor Daqu with different storage periods [36]. For bacterial communities, RDA explained 85.18% of the total variation between the bacterial genera and physicochemical indexes, with RDA1 and RDA2 accounting for 76.5% and 8.68%, respectively (Figure 4A and Figure 4B). Starch and moisture were positively correlated with *Saccharopolyspora*, *Kroppenstedtia*, *Virgibacillus*, and *Bacillus*, but negatively correlated with *Oceanobacillus*. Acidity, saccharification power, fermentation power, and acidic protease activity showed positive correlations with *Bacillus*, *Kroppenstedtia*, and *Oceanobacillus*, while being negatively correlated with *Saccharopolyspora* and *Virgibacillus*. For fungal communities, RDA explained 79.48% of the total variation (RDA1: 51.94%, RDA2: 27.54%). Starch, moisture, and acidity were positively correlated with *Byssochlamys*, but negatively correlated with *Thermomyces*, *Aspergillus*, and *Saccharomycopsis*. Fermentation power was positively correlated with *Thermomyces* and *Aspergillus*, while saccharification power and acidic protease activity were positively associated with *Thermomyces* and *Byssochlamys*. A positive correlation between the average relative abundance of *Bacillus* and the saccharification capacity of Daqu was observed by Gan et al. [19]. They identified α-glucosidase as the main pathway in the saccharification process, among which *Bacillus* is the primary microorganism for the gene expression of α-glucosidase. Moreover, many studies have indicated that *Bacillus* can secrete significant levels of amylase and protease—enzymes that play an essential role in the processes of liquefaction and saccharification [41]. Studies have shown that *Virgibacillus* can significantly increase the concentrations of 10 pyrazine compounds and 3 nitrogen-containing heterocyclic compounds by synthesizing proteases. Additionally, it can participate in pyruvate metabolism and tetramethylpyrazine metabolism through glyceraldehyde phosphate dehydrogenases (EC: 2.7.9.2, EC: 2.7.1.40) [42]. As a dominant functional bacterium in high-temperature Daqu, *Oceanobacillus* can produce various enzymes, such as cellulase and neutral proteinase, and mainly participates in the metabolism to generate the key volatile flavor substances of Baijiu, including pyrazines and phenols [43].

The Spearman correlation of microbial communities and volatile flavor substances *Bacillus* exhibited a strong positive correlation with key pyrazines (2,3,5,6-tetramethylpyrazine, 2,3,5-trimethylpyrazine) and 3-hydroxy-2-butanone (Figure 4C,D). *Lactobacillus* showed positive correlations with multiple volatile compounds, while *Saccharomyces* was positively correlated with alcohols (butanol, 1-pentanol, isoamyl alcohol) but negatively correlated with most other alcohols [25]. *Thermoascus* and *Thermomyces* were positively correlated with most esters, which are critical for the aroma of Jiang-flavor Baijiu [26]. Additionally, *Aspergillus* is regarded as playing a pivotal role in saccharification and fermentation during Jiang-flavor Baijiu manufacturing [44,45]. It is capable of producing multiple extracellular enzymes, such as acidic/alkaline proteases, which in turn leads to the generation of a substantial quantity of secondary metabolites [9,46]. While 4-ethylguaiacol is typically associated with Brettanomyces in wine fermentation, its occurrence in Daqu originates from alternative biosynthetic pathways. Isolated Bacillus species exhibit feruloyl esterase activity, which mediates the release of ferulic acid; this compound is subsequently converted to 4-ethylguaiacol [47]. *Thermophilic* fungi such as *Thermomyces* also contribute to the production of these phenolic compounds via lignin degradation. The absence of Brettanomyces in our sequencing data (detection limit ~0.01%) validates these alternative pathways, which is consistent with the 50–60 °C environment of Jiang-flavor Daqu production—exceeding the maximum growth temperature (35 °C) of Brettanomyces [48].

### 3.5. Differences in Physicochemical Properties and Volatile Substances of Jiang-Flavor Daqu with Different Storage Periods During the Brewing Stage

To explore the differences in physicochemical properties and volatile substances during brewing induced by Jiang-flavor Daqu with different storage periods (0, 30, 60, 90, 120, 150, 180 days), simulated brewing experiments were conducted, and physicochemical indexes of fermented grains and volatile compounds in the base wine were analyzed.

**Figure 5:**
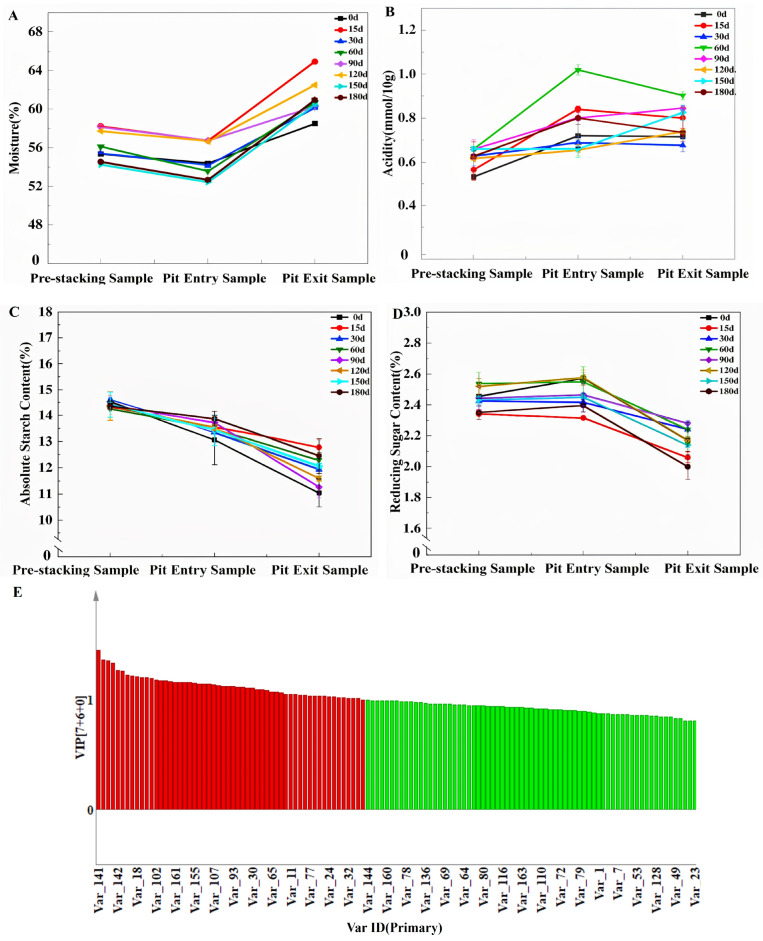
Influence of Daqu fermentation at different storage stages. (**A**) Moisture and (**B**) acidity in the brewing stages of Jiang-flavor Daqu in different storage periods. (**C**) Starch and (**D**) reducing sugars in the brewing stage of Jiang-flavor Daqu at different storage periods. (**E**) VIP diagram of important metabolites.

The brewing process exhibited dynamic physicochemical changes. The moisture content initially decreased, which was likely due to microbial consumption during pile-fermentation, then increased in later stages, possibly from microbial autolysis and acidogenic metabolism (Figure 5A). Acidity rose during pile-fermentation, as microbes proliferated but stabilized or slightly decreased afterwards, which was potentially inhibited by high ethanol levels or esterification reactions (Figure 5B). Starch content declined consistently, with a rapid decrease early in fermentation facilitated by amylases slowing later, possibly due to feedback inhibition from glucose accumulation (Figure 5C). Concurrently, reducing sugars accumulated initially from rapid starch hydrolysis, then decreased as they were consumed by residual microbes for growth and conversion to ethanol (Figure 5D). These shifts reflect the complex microbial activity and biochemical transformations that are essential for Jiang-flavor Baijiu production.

HS-SPME-GC-MS identified 68 volatile compounds in the base wine, including 21 esters, 15 alcohols, 10 pyrazines, 8 acids, and 14 others. The Daqu storage period significantly affected the volatile composition: base wine with 0–60-day Daqu had a higher alcohol content (e.g., isoamyl alcohol, 1.82–2.15 mg/L) and short-chain fatty acids (e.g., acetic acid, 0.95–1.12 mg/L) (Figure 5E and Table S2). In contrast, wine with 120–180-day Daqu showed higher ester (e.g., ethyl caproate, 1.56–1.88 mg/L) and pyrazine (e.g., 2,3,5-trimethylpyrazine, 0.78–0.92 mg/L) contents—key contributors to the mellow aroma of Jiang-flavor Baijiu [49]. PLS-DA identified 18 marker compounds (VIP > 1), including ethyl caproate and 2,3,5-trimethylpyrazine, which effectively distinguished the base wine brewed with Daqu of different storage periods (Figure S4).

### 3.6. Untargeted Metabolomics Analysis of Jiang-Flavor Daqu During Storage and Brewing Stages

Untargeted metabolomics based on liquid chromatography–mass spectrometry (LC-MS) was employed to analyze the metabolic differences in Jiang-flavor Daqu across different storage periods (0, 30, 60, 90, 120, 150, 180 days) and clarify the metabolic regulatory mechanisms underlying Daqu quality formation.

**Figure 6:**
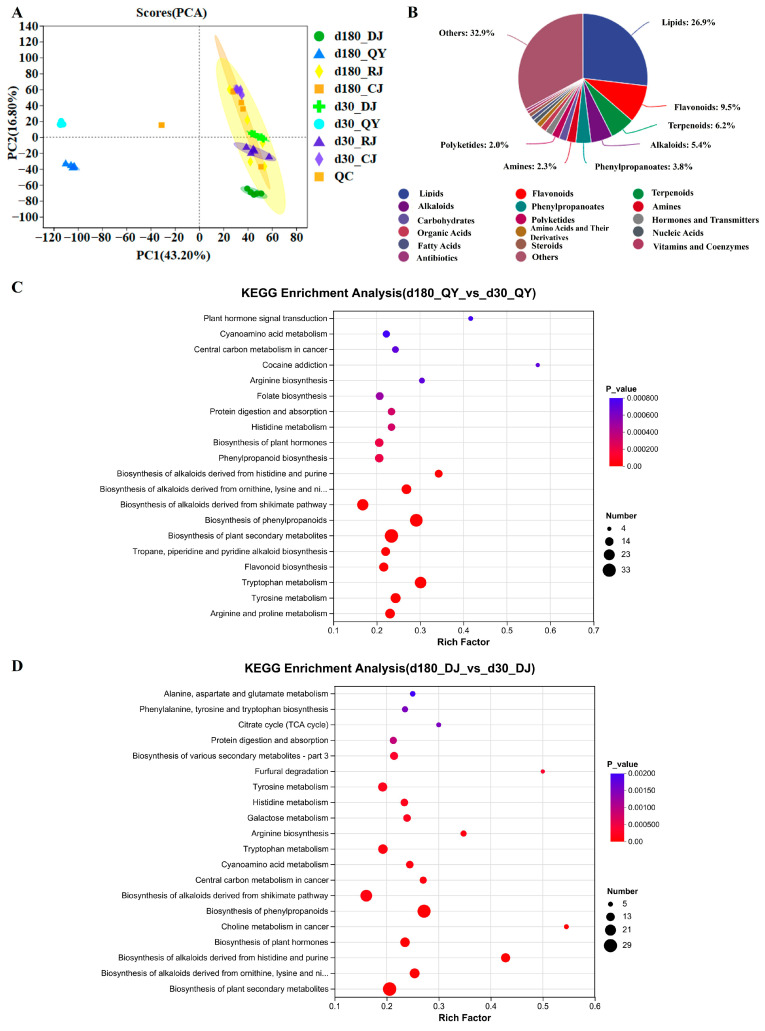
Untargeted metabolomics analysis of Jiang-flavor Daqu during the storage and brewing stages. (**A**) Storage for 30 d and 180 d analysis of principal components of non-volatile metabolites in the storage and brewing stages of Jiang-flavor Daqu. (**B**) Storage for 30 d and storage for 180 d pie chart of the overall distribution of non-volatile metabolites in the storage and brewing stages of Jiang-flavor Daqu. (**C**) Storage of 30 d vs. storage of 180 d koji drug. (**D**) 30 d before stacking vs. 180 d before stacking zaopei.

The results of the principal component analysis (PCA) indicated a trend of metabolite separation between groups, suggesting that there were differences in metabolites among the various sample groups (Figure 6A). A total of 1882 non-volatile metabolites were identified from 40 samples, including 506 lipids, 178 flavonoids, 117 terpenoids, 102 alkaloids, 71 phenylpropanoids, 44 amines, 37 carbohydrates, 37 polyketides, 33 hormones and transmitters, 29 organic acids, 25 amino acids and their derivatives, 22 nucleic acids, 19 fatty acids, 18 steroids, 14 vitamins and coenzymes, 11 antibiotics, and 619 others (Figure 6B). To evaluate the reliability of metabolic profiling and group separation, orthogonal partial least squares discriminant analysis (OPLS-DA) was performed. The OPLS-DA score plot showed clear separation among Daqu samples with different storage periods, with no overlap between groups (Figure S5). The model validation results (R^2^X = 0.892, R^2^Y = 0.915, Q^2^ = 0.876) indicated good fitting and predictive ability, confirming distinct metabolic profiles driven by storage duration (Table S3).

Differential metabolites were screened using strict criteria: variable importance in projection (VIP) > 1.15, *p* < 0.001, and fold change (FC) ≥ 1.5 (up-regulation) or FC ≤ 0.67 (down-regulation) [9,50]. Differential metabolites were also screened via T-test (VIP > 1, *p* < 0.01). For 30-day vs. 180-day Jiang-flavor Daqu, 28 differential metabolites were identified in Daqu samples during storage and brewing, 65 in pre-stacking samples, 79 in pit-entry samples, and 52 in pit-exit samples (Figure S6).

All differential metabolites in each group were matched to the KEGG database, and pathway information related to these metabolites was obtained [51]. Enrichment analysis was performed on the top 20 metabolic pathways ranked by *p*-value in each comparison group, yielding pathways with high differential metabolite enrichment. In the d180QY vs. d30QY group, differential metabolites were mainly enriched in metabolic pathways, furfural degradation, phenylpropanoid biosynthesis, and flavonoid biosynthesis (Figure 6C). The d180DJ vs. d30DJ group showed enrichment in metabolic pathways, furfural degradation, flavone and flavonol biosynthesis, and tropane, piperidine, and pyridine alkaloid biosynthesis (Figure 6D). For the d180RJ vs. d30RJ and d180CJ vs. d30CJ groups, the key enriched pathways included metabolic pathways, furfural degradation (only d180RJ vs. d30RJ), flavone and flavonol biosynthesis, betalain biosynthesis, and flavonoid biosynthesis, with d180CJ vs. d30CJ additionally involving Phenylpropanoid biosynthesis and pantothenate and CoA biosynthesis (Figure S7). Notably, pre-stacking, pit-entry, and pit-exit stages shared more consistent enriched pathways, which were mostly related to flavonoid and flavonol synthesis, while Daqu storage-related pathways primarily involved phenylic acid, flavonoid biosynthesis, and furfural degradation. Overlapping pathways may be critical for the storage and brewing stages. Notably, flavonoid biosynthesis and tyrosine metabolism showed the highest enrichment degrees, suggesting that they are core metabolic pathways regulating Daqu quality during storage. Amino acids are important taste compounds among the non-volatile components of Jiang-flavor Baijiu and possess various biological activities. Furthermore, Daqu ferments at high temperatures, which accelerates the decomposition of proteins and amino acids, and promotes the Maillard reaction to produce important flavor components such as furfuryl alcohol, pyrazines, and aromatic compounds. Meanwhile, amino acids are used as substrates by lactic acid bacteria and yeasts in Daqu, thereby enhancing their esterase activity and generating more ester flavor compounds. Thus, amino acids and their derivatives are important metabolites that affect the quality of Daqu. In addition to amino acids and their derivatives, there are significant differences in lipid metabolites among the samples. Lipids are esters, and their derivatives are composed of fatty acids and alcohols. These substances can form a variety of products through oxidation and hydrolysis, and serve as a source of flavor compounds in Jiang-flavor Baijiu.

Additional analysis of metabolite dynamics across storage stages revealed that the amino acid content was significantly higher in the early storage stage (0–60 days) (e.g., tyrosine content peaked at 30 days with 2.87 μg/g) and gradually decreased in the late stage (90–180 days). In contrast, flavonoids and their derivatives (e.g., naringenin, quercetin) accumulated continuously, with contents at 180 days being 3.2-fold higher than those at 0 days. These trends indicated that storage duration drives the shift in Daqu metabolism from amino acid accumulation to flavonoid synthesis, which contributes to the improved antioxidant capacity and flavor stability of Daqu [10]. Pre-stacking, pit entry, and pit exit have more uniform enriched metabolite pathways, most of which are related to the synthesis of flavonoids and flavonoid-like compounds, while the enriched pathways in Daqu sample storage are mainly associated with the biosynthesis of phenolic acids and flavonoids, as well as the degradation of furfural [16]. Overlapping metabolic pathways may be the key metabolic pathways in the storage and brewing stages. Daqu mainly metabolizes amino sugars, which also serves as a survival strategy for microorganisms under oligotrophic conditions [8]. This indicates that the directly available carbohydrates in Daqu can hardly promote microbial reproduction in the late storage stage, and Daqu thus enters a steady state.

## 4. Conclusions

This study takes Jiang-flavor Daqu from a certain enterprise in Sichuan Province as the research object, aiming to explore its characteristics during the 0–180-day storage period and the relevant impacts on liquor-making. The results show that the saccharifying power and fermenting power of Daqu first increased and then stabilized, reaching 205 mg/g·h and 0.71 g/g·72 h, respectively, at 180 days. The microbial diversity first rose and then declined, with *Virgibacillus* and *Oceanobacillus* alternating as the dominant bacteria. The flavor substances were more abundant within 60 days of storage, and the content of pyrazines was the highest at 180 days. The brewing stage had the greatest impact on metabolites, and flavonoid synthesis was the key pathway. This study provides a basis for the scientific storage of Daqu and the standardization of Baijiu quality.

## Figures and Tables

**Table 1:** Analysis of microbial community diversity of Jiang-flavor Daqu in different storage periods.

Sample	Coverage		ACE		Chao1		Simpson		Shannon	
ID	Fungi	Bacteria	Fungi	Bacteria	Fungi	Bacteria	Fungi	Bacteria	Fungi	Bacteria
0 d	0.99>	0.99>	67.02	138.16	68.21	133.58	0.75	0.20	0.62	2.18
15 d	0.99>	0.99>	72.23	264.47	66.73	247.64	0.27	0.26	1.48	2.05
30 d	0.99>	0.99>	75.60	192.13	72.50	190.75	0.23	0.28	1.70	1.76
60 d	0.99>	0.99>	89.74	265.75	86.90	261.83	0.35	0.12	1.26	2.68
90 d	0.99>	0.99>	87.42	300.46	83.07	295.55	0.32	0.13	1.51	2.70
120 d	0.99>	0.99>	87.45	431.77	87.54	430.88	0.39	0.09	1.25	2.90
150 d	0.99>	0.99>	128.50	282.66	131.07	267.31	0.26	0.36	1.64	1.62
180 d	0.99>	0.99>	105.20	170.23	104.29	169.53	0.19	0.23	2.25	2.09

## Data Availability

The original contributions presented in this study are included in the article and supplementary material. Further inquiries can be directed to the corresponding authors.

## Supplementary Materials

The following supporting information can be downloaded at: https://www.mdpi.com/article/10.3390/foods15020220/s1, Figure S1. Hierarchical clustering tree analysis of bacterial communities (A) and fungal communities (B) of Jiang-flavor Daqu at different storage periods. Figure S2. (A)Relative abundance of bacterial communities at the phylum level of Jiang-flavor Daqu at different storage periods. (B) Relative abundance of fungal communities at the phylum level of Jiang-flavor Daqu at different storage periods. Figure S3. Heat map of volatile flavor content of Jiang-flavor Daqu during different storage periods. Figure S4. OPLS-DA score of volatile flavor substances in the brewing stage of Jiang-flavor Daqu at different storage stages. Figure S5. Based on orthogonal partial least squares analysis (OPLS-DA) analysis of the differences between the storage and brewing stages of the Jiang-flavor Daqu that were stored for 30 days and 180 days: (A) Storage of 30 d vs. storage of 180 d koji drug. (B) 30 d before stacking vs. 180 d before stacking zaopei. (C) 30 d in zaopei vs. 180 d in zaopei. (D) 30 d out of the zaopei vs. 180 d out of the zaopei. Figure S6. Volcanic diagram of differential metabolites of Jiang-flavor Daqu at the storage and brewing stages of 30 d and 180 d: (A) Storage of 30 d vs. storage of 180 d koji drug. (B) 30 d before stacking vs. 180 d before stacking zaopei. (C) 30 d in zaopei vs. 180 d in zaopei. (D) 30 d out of the zaopei vs. 180 d out of the zaopei. Figure S7. KEGG enrichment bubble plot of Jiang-flavor Daqu at 30 d and 180 d during the storage and brewing stages. (A) 30 d in zaopei vs. 180 d in zaopei. (B) 30 d out of the zaopei vs. 180 d out of the zaopei. Table S1. Volatile flavor substances (VIP value > 1) in Jiang-Flavor Daqu with different storage periods. Table S2. Names of Key Compounds in the Impact of Daqu with Different Storage Periods on Fermentation (VIP value > 1). Table S3. OPLS-DA model evaluation parameters.
