# Supplementary material for: Evolution of Jiang-Flavor Daqu’s Characteristics During Different Storage Stages and Influence on Simulated Brewing Fermentation
Source: Foods. 2026 Jan 8;15(2):220. doi: 10.3390/foods15020220 (PMC12840205; doi:10.3390/foods15020220)
Supplement: Supplementary file 1 [file foods-15-00220-s001.zip › foods-4031296-supplementary.pdf]

Evolution of Jiang-flavor Daqu's Characteristics During Different Storage Stages and Influence on Simulated Brewing Fermentation

Zihan Chen[1], Han Wang [1], Chongchao Wu [1], Xing Zheng [1], Guida Zhu[1], Jing Yu[1], Qiuxiang Tang[1,*], Ping Song[1, *]

[1] State Key Laboratory of Microbial Technology, School of Food Science and Pharmaceutical Engineering, Nanjing Normal University, Nanjing 210023, P.R. China

Corresponding author: Qiuxiang Tang *, Ping Song

Email: tangqiuxiangtqx@163.com; songping@njnu.edu.cn

Tel & Fax: +86-25-85898687

Address: Xuelin Road, Nanjing, 210046, P.R. China

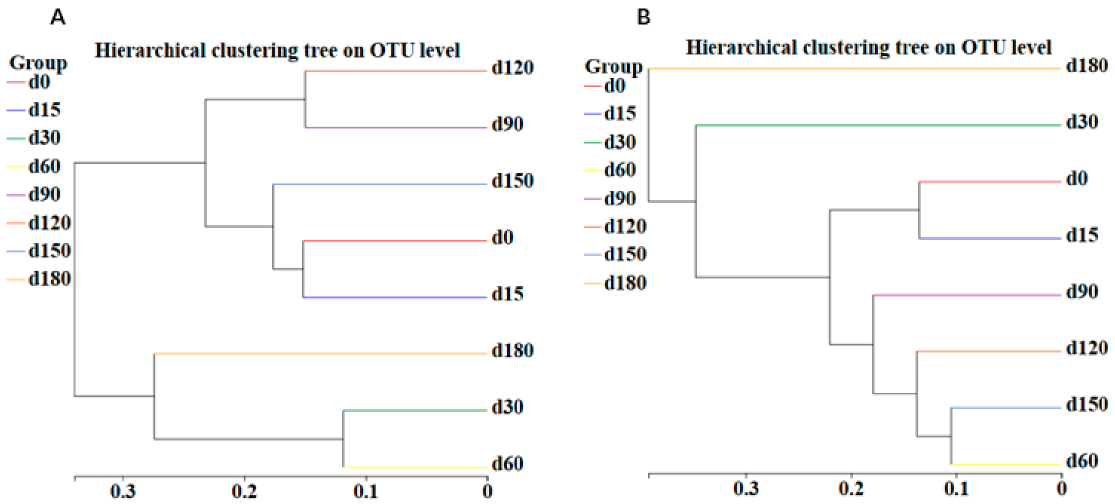

Figure S1 Hierarchical clustering tree analysis of bacterial communities (A) and fungal communities (B) of Jiang-flavor Daqu at different storage periods

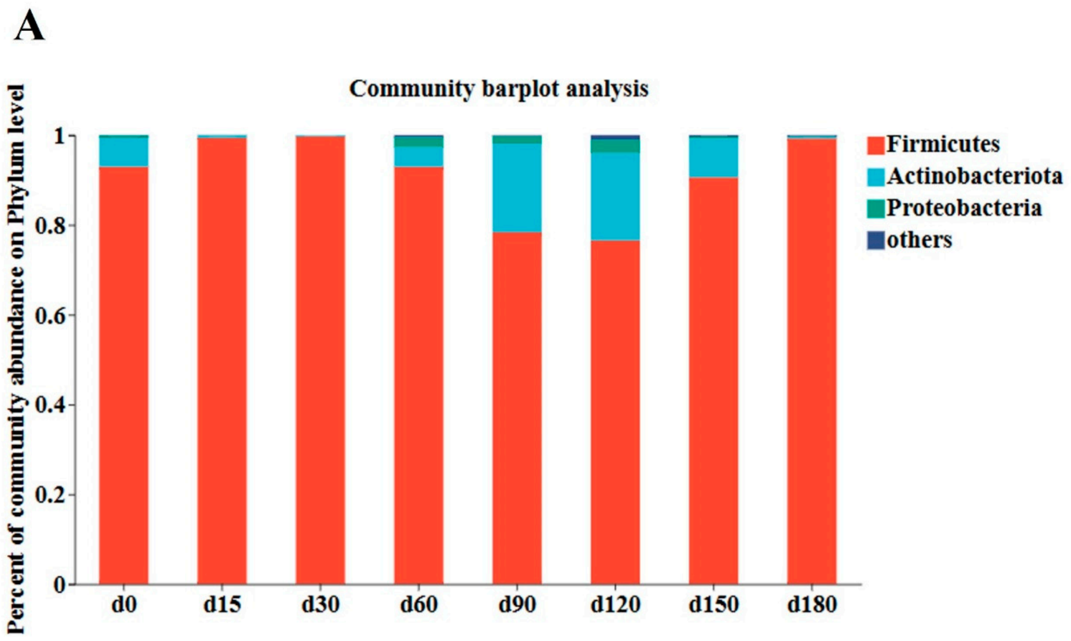

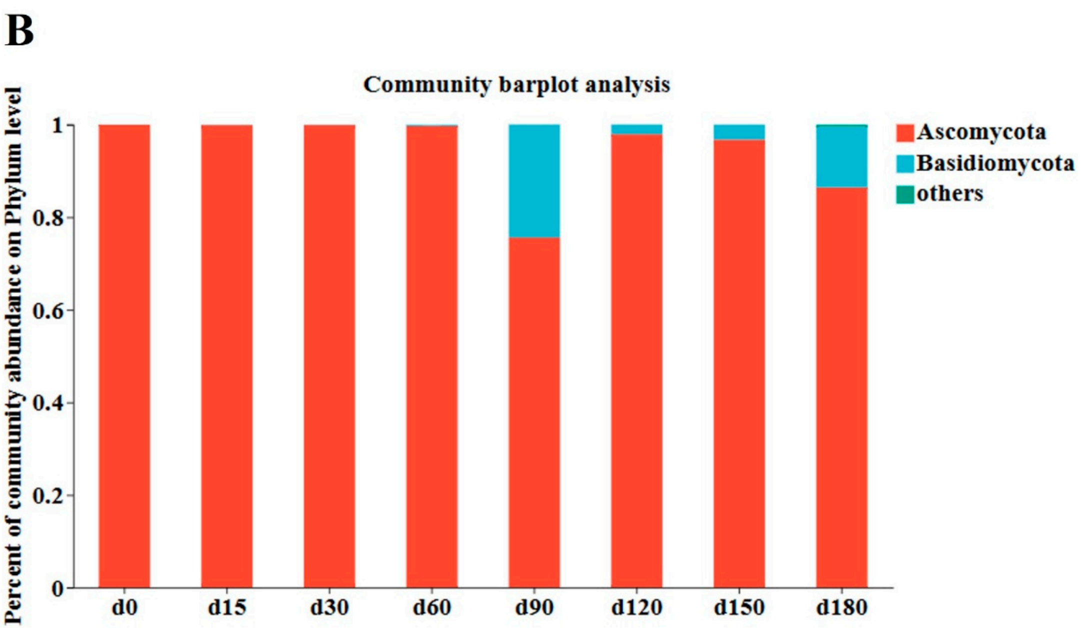

Figure S2 (A)Relative abundance of bacterial communities at the phylum level of Jiang-flavor Daqu at different storage periods. (B) Relative abundance of fungal communities at the phylum level of Jiang-flavor Daqu at different storage periods.

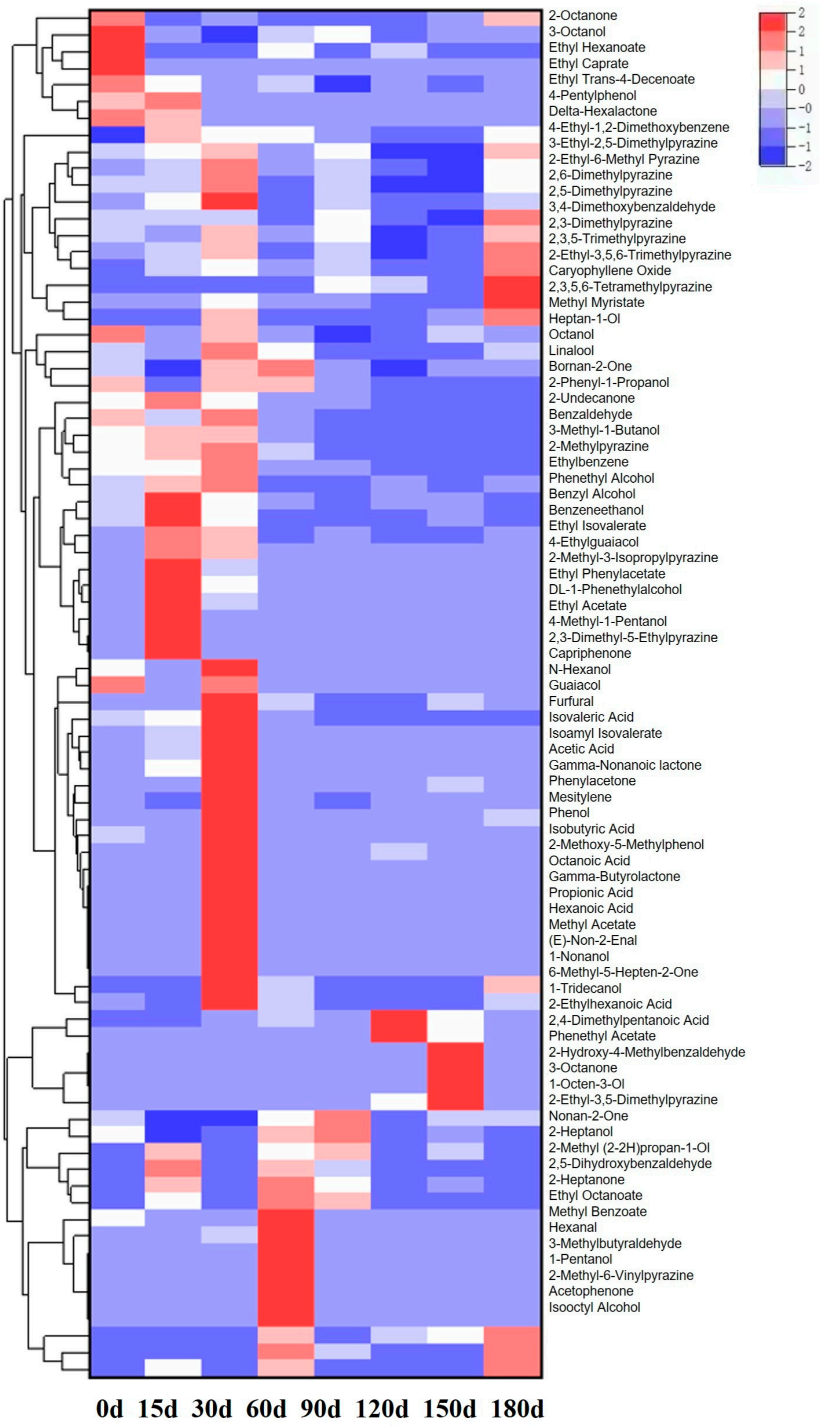

Figure S3 Heat map of volatile flavor content of Jiang-flavor Daqu during different storage periods.

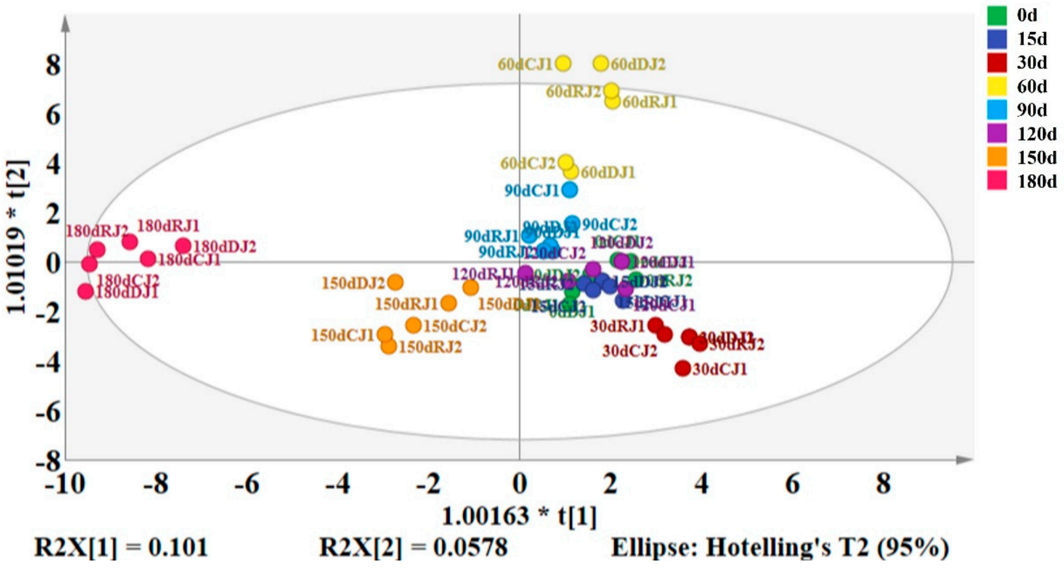

Figure S4 OPLS-DA score of volatile flavor substances in the brewing stage of Jiang-flavor Daqu at different storage stages.

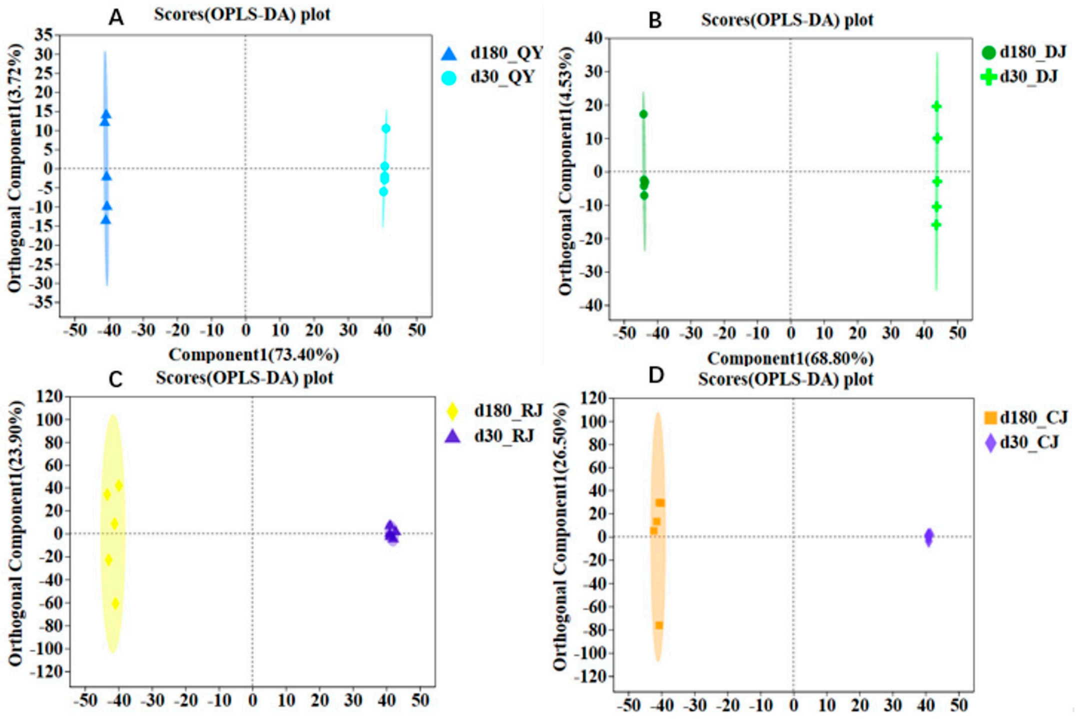

Figure S5 Based on orthogonal partial least squares analysis (OPLS-DA) analysis of the differences between the storage and brewing stages of the Jiang-flavor Daqu that were stored for 30 days and 180 days：(A) Storage of 30 d VS storage of 180 d koji drug. (B) 30 d before stacking VS 180 d before stacking zaopei. (C) 30 d in zaopei VS 180 d in zaopei. (D) 30 d out of the zaopei VS 180 d out of the zaopei.

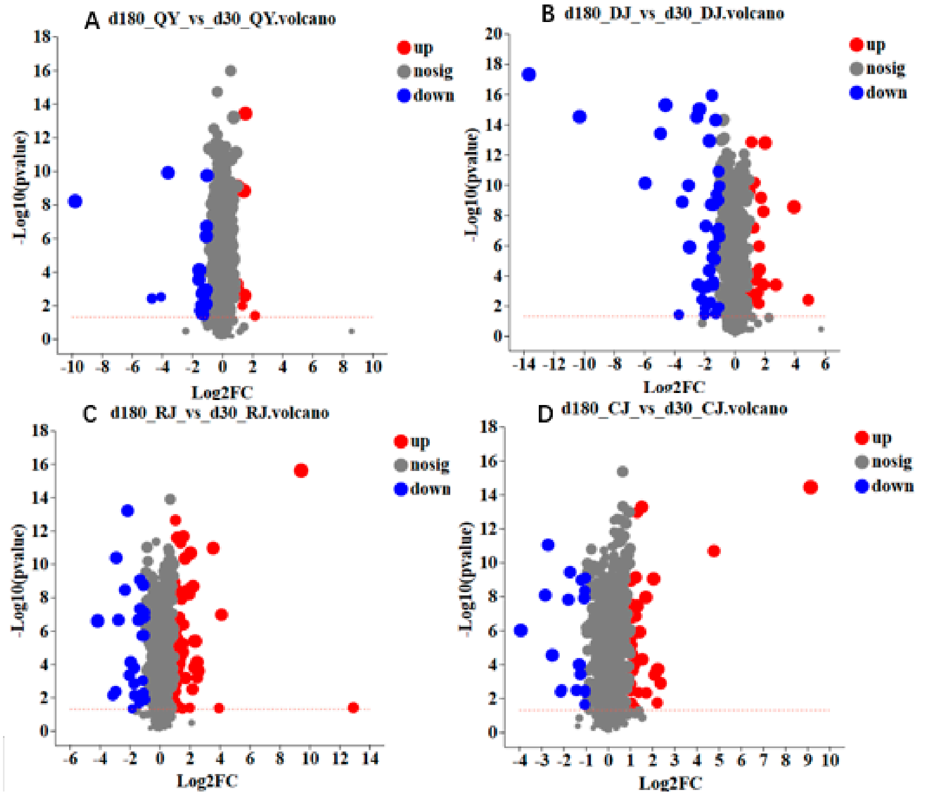

Figure S6 Volcanic diagram of differential metabolites of Jiang-flavor Daqu at the storage and brewing stages of 30 d and 180 d：(A) Storage of 30 d VS storage of 180 d koji drug. (B) 30 d before stacking VS 180 d before stacking zaopei. (C) 30 d in zaopei VS 180 d in zaopei. (D) 30 d out of the zaopei VS 180 d out of the zaopei.

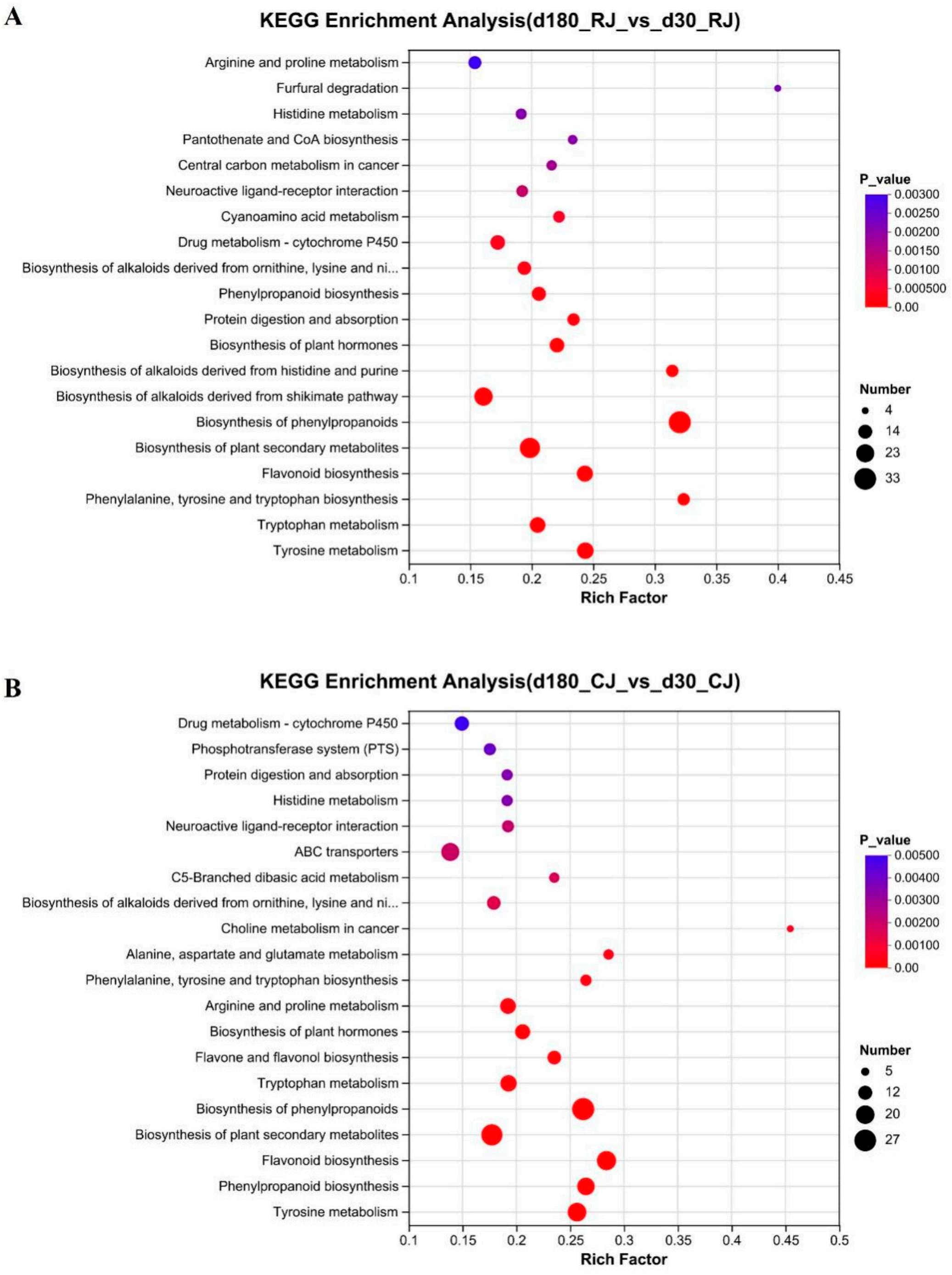

Figure S7 KEGG enrichment bubble plot of Jiang-flavor Daqu at 30 d and 180 d during the storage and brewing stages. (A) 30 d in zaopei VS 180 d in zaopei. (B) 30 d out of the zaopei VS 180 d out of the zaopei.

Table S1 Volatile flavor substances (VIP value >1) in Jiang-Flavor Daqu with different storage periods

Var ID (Primary)	Names	VIP value
Var_75	4-ethylguaiacol	1.42437
Var_51	2-Phenyl-1-propanol	1.40541
Var_56	Ethyl phenylacetate	1.40014
Var_72	4-Pentylphenol	1.39534
Var_53	Phenethyl alcohol	1.37883
Var_52	Benzyl alcohol	1.36221
Var_60	2-Isopropyl-3-methylpyrazine	1.33846
Var_9	(E)-non-2-enal	1.27876
Var_38	Phenylacetone	1.23068
Var_18	Ethyl isovalerate	1.22379
Var_2	1-Nonanol	1.21539
Var_21	Methyl Myristate	1.20445
Var_55	Benzaldehyde	1.19981
Var_36	Nonan-2-one	1.17623
Var_13	delta-Hexalactone	1.17418
Var_50	Benzeneethanol	1.16965
Var_54	Gamma-nonanoic lactone	1.16243
Var_45	Isopentanol	1.12946
Var_73	2-Methoxy-5-methylphenol	1.12752
Var_69	2-methylpyrazine	1.11218
Var_25	Isobutyric Acid	1.11075
Var_32	Phenol	1.11055
Var_82	Mesitylene	1.10866
Var_44	Ethylbenzene	1.10702
Var_74	Guaiacol	1.08042

Var_41	Furfural	1.06228
Var_29	Isovaleric acid	1.059
Var_58	Acetic acid	1.05059
Var_65	2,5-dimethylpyrazine	1.0454
Var_17	Isoamyl isovalerate	1.04176
Var_59	2,3-Dimethyl-5-ethylpyrazine	1.03731
Var_47	N-Hexanol	1.00982

Table S2 Names of Key Compounds in the Impact of Daqu with Different Storage Periods on Fermentation (VIP value >1)

Var ID (Primary)	Names	VIP value
Var_141	2-Undecanone	1.45245
Var_21	Tridecyl alcohol	1.36314
Var_129	2-Methoxy-4-Methyl Phenol	1.35597
Var_47	Isovaleric acid	1.33788
Var_142	2-Tridecanone	1.27166
Var_70	Methyl palmitate	1.22483
Var_127	4-Ethylphenol	1.22095
Var_18	butane-2,3-diol	1.2081
Var_137	Geranylacetone	1.20616
Var_154	Alpha-Humulene	1.2046
Var_92	Ethyl isovalerate	1.19931
Var_102	Ethyl undecanoate	1.17974
Var_76	Ethyl elaidate	1.17399
Var_81	Ethyl acetate	1.17366

Var_143	Fitone	1.16434
Var_161	Veratrole	1.16359
Var_126	4-ethylguaiacol	1.16192
Var_3	3-Methyl-2-buten-1-ol	1.16178
Var_16	Geraniol	1.15763
Var_155	(E)- β -Farnesene	1.14955
Var_91	Ethyl 2-methylbutyrate	1.14706
Var_22	4-Methyl-1-pentanol	1.14677
Var_46	Isobutyric Acid	1.14519
Var_107	3-oxobutan-2-yl 2-methylbutanoate	1.13524
Var_130	2,4-Di-tert-butylphenol	1.13144
Var_139	Acetophenone	1.12637
Var_151	B-Bisabolene	1.12427
Var_93	3-Methylbutyl 2-methyl butanoate	1.12067
Var_10	Benzyl alcohol	1.11962
Var_74	Methyl oleate	1.11292
Var_97	Ethyl butanoate	1.10848
Var_30	Nonyl aldehyde	1.10782
Var_2	Isopentanol	1.0976
Var_45	Acetic acid	1.09117
Var_20	:β-Eudesmol	1.08925
Var_100	Ethyl benzoate	1.07094
Var_66	Ethyl phenylacetate	1.06405
Var_37	Furfural	1.05238
Var_11	Phenethyl alcohol	1.05206

Var_59	Isoamyl acetate	1.04863
Var_150	(-)-Aristolene	1.04542
Var_96	Monoethyl succinate	1.03998
Var_77	Methyl linoleate	1.03971
Var_8	Trans-2-Octen-1-ol	1.03573
Var_145	Geranylacetone	1.03353
Var_24	Palustrol	1.02868
Var_124	Guaiacol	1.02606
Var_105	Isoamyl isobutyrate	1.02214
Var_131	2,3,5-Trimethylpyrazine	1.01837
Var_32	1-Decanal	1.01312
Var_87	9,12,15-Octadecatrienoic acid, methyl ester	1.01129
Var_62	Ethyl Octanoate	1.01074
Var_125	Phenol	1.0001

Table S3 OPLS-DA model evaluation parameters

Number	Comparison Group	R2X	R2Y	Q2
A	d180_QY VS d30_QY	0.772	1	0.996
B	d180_DJ VS d30_DJ	0734	1	0.995
C	d180_RJ VS d30_RJ	0.790	0.999	0.992
D	d180_CJ VS d30_CJ	0.829	1	0.996
